# What’s in a Hub?—Representing Identity in Language and Mathematics

**DOI:** 10.1016/j.neuroscience.2020.02.032

**Published:** 2020-02-27

**Authors:** Aditi Arora, Belinda Pletzer, Markus Aichhorn, Josef Perner

**Affiliations:** Centre for Cognitive Neuroscience, Department of Psychology, University of Salzburg, 5020 Salzburg, Austria

**Keywords:** fMRI, hubs, parietal cortex, precuneus, metacognition

## Abstract

Hubs emerge in structural and resting state network analysis as areas highly connected to other parts of the brain and have been shown to respond to several task domains in functional imaging studies. A cognitive explanation for this multi-functionality is still wanting. We propose, that hubs subserve domain-general meta-cognitive functions, relevant to a variety of domain-specific networks and test this hypothesis for the example of processing explicit identity information. To isolate this meta-cognitive function from the processing of domain-specific context, we investigate the overlapping activations to linguistic identity processes (e.g. Mr. Dietrich is the dentist) on the one hand and numerical identity processes (e.g. do “3 × 8” and “36–12” give the same number) on the other hand. The main question was, whether these overlapping activations would fall within areas, consistently identified as hubs by network-based analyses. Indeed, the two contrasts showed significant conjunctions in the left inferior parietal lobe (IPL), precuneus (PC), and posterior cingulate. Accordingly, identity processing may well be one domain-general meta-cognitive function that hub-areas provide to domain-specific networks. For the parietal lobe we back up our hypothesis further with existing reports of activation peaks for other tasks that depend on identity processing, e.g., episodic recollection, theory of mind, and visual perspective taking.

## Introduction

Much of cognitive neuroscience focuses on tracing the neural substrate of common sense cognitive domains, e.g., the social brain (Brothers, 1990; Frith and Frith, 2010), the linguistic brain (Fedorenko and Kanwisher, 2009; Blank and Fedorenko, 2017), the number sense (Dehaene, 2011). This work is corroborated by resting state and task-based connectivity analyses demonstrating a very robust distinction of different brain networks subserving different cognitive functions (Biswal et al., 2010; Laird et al., 2011; Smith et al., 2009). Thereby, remarkable overlap has been observed between intrinsic-connectivity networks derived from resting-state and task-based data (Biswal et al., 2010; Laird et al., 2011; Smith et al., 2009; Rasero et al., 2018; Kieliba et al., 2019; Fong et al., 2019; Alexander-Bloch et al., 2018). It has also been observed that changes in functional network dynamics are usually preceded by changes in the structural connectome, this linking structural and functional brain networks (Zuo et al., 2017). However, these networks cannot operate completely independently. Structural and functional network analyses robustly show that a certain class of areas interconnect different networks (Utevsky et al., 2014; van den Heuvel and Sporns, 2011; Power et al., 2013; de Pasquale et al., 2012; Hagmann et al., 2008). These areas include the posterior cingulate cortex (PCC)/precuneus (PC), inferior and superior parietal areas, thalamus, hippocampus, putamen and superior frontal gyrus, which emerge with high consistency as hubs across different imaging modalities (van den Heuvel and Sporns, 2011). Particularly, the PCC, PC, paracentral lobule, as well as superior and inferior parietal cortex together emerged as structural “core” (s-core) of the human cerebral cortex (Hagmann et al., 2008). They constitute connector hubs that link all major modules of the human cerebral cortex. However, while a consensus exists on which areas form the “hubs” that interconnect different brain networks, the functional role of these hubs remains yet to be uncovered (Humphreys and Lambon Ralph, 2015). Is their role simply to pass information from one network to another or do they perform certain cognitive functions. If the letter is the case, a plausible approach would be that hubs subserve domain-general cognitive functions, which are relevant to all cognitive domains. Accordingly, each network would be able to draw on these nodes whenever such a domain general function is required, which would explain why hubs respond to such a large variety of different contents. Such a situation would be more efficient than each network providing said know-how itself. To answer this question, a first step is to identify candidates for such domain-general cognitive functions. In a second step, it has to be assessed whether these functions can be pinpointed to hub areas, i.e. whether their activations can be found in hub areas rather than elsewhere in the brain. In order to achieve that, domain-general cognitive functions need to be isolated from the domain-specific context in which they are embedded via proper experimental designs. In the current manuscript we pursue the case of identity processing as a candidate for such a domain-general cognitive function that hub areas may provide.

Explicit representation of identity requires higher order meta-cognitive processing beyond the basic level of information about a network’s domain. In the following we outline, why identity processing is relevant to various brain networks including the social brain (e.g. recollecting the identity of a familiar looking person), the linguistic brain (e.g. processing identity statements: “The dentist is Mr. Dietrich”), and the numerical brain (e.g. 32–12 yields the identical number to 3 × 8). To do so, we need to distinguish two levels of dealing with identity. Basic identity processing is a necessity for any intelligent system that tracks individuals over time and gains knowledge about them. The most familiar way of achieving this is to represent the individual at every encounter with the same mental symbol.^1^ This is the basic form of trading on identity (Campbell, 1987), or identity *de jure* (Recanati, 2014), where the laws of how the system works determine identity. This provides a procedural (implicit) and automatic grasp of identity and every network has to have its method of doing this, and methods might differ for different networks. An independent expert processor would seem superfluous. However, there are cases where this basic, automatic way of consistently using the same symbol for an individual does not suffice. Sometimes identity needs to be represented explicitly.

Frege (1967) was puzzled by the fact that identity statements like, “The Evening Star is the Morning Star,” can be informative to the ancient Babylonians who thought that these were two different stars, when in fact they both refer to the planet Venus. This is a puzzle because in usual predicative sentences, e.g., “The Morning Star is bright in the morning,” which predicates of Venus being bright in the morning, the statement remains informative when one rephrases it using its other name: “The Evening Star is bright in the morning.” This indicates that the referent, Venus, matters but not any particular way of referring to it. Not so for the identity statement. If we replace one name for the other, it turns into a completely uninformative, trivial piece of wisdom: “The Evening Star is the Evening Star”. The reason for this loss of informativeness is that basic identity processing already treats “Evening Star” as referring to the same entity on both occasions, whereas “Evening Star” and “Morning Star” are taken to refer to different things unless explicit identity information “The Evening Star is the Morning Star” is provided.

The same observation can be made with numbers. The informative equation “2 × 4 = 8” expresses the identity of the number denoted by the left side formula with the number on the right side. As in the case of “The Evening Star is the Morning Star,” replacing one side with the expression on the other side makes the equation uninformative: “8 = 8”. Whereas predicative information about the number 8, e.g., “(10 – 2) > 7” stays informative: “8 is bigger than 7.”

From these considerations it follows that identity statements play a special role. Normal, predicative statements provide information to be processed by its network. Whereas, identity statements provide meta-cognitive/-representational information. They tell each network of how its representations that conceptualise an object in different ways (Venus as Morning or as Evening Star; the number 8 by the expression “2 × 4” or the numeral “8”) can be treated in the same way. Thus, identity statements provide metarepresentational/meta cognitive information for each network, and it may be economical to have a set of brain regions that do this special work for different networks. To assess the likelihood of the existence of such regions for linguistic statements and mathematical equations we look for potential overlap of brain activations for these tasks in existing imaging data.

### Identity statements in the brain

An existing study showed that the left inferior parietal lobe (IPL) and the PC were more strongly activated for identity statements “The lawyer is Mr Moser” than for predicative statements “The lawyer is young” (Arora et al., 2015). Several studies investigating the neuroanatomical correlates of anaphoric reference (Nieuwland et al., 2007) point to similar regions: bilateral activation of lateral and medial parietal regions when faced with referential ambiguity (“Ronald told Frank that **he** had a positive…”) or referential failure (“Rose told Emily that **he** had a positive…”) in contrast with referential coherence (“Ronald told Emily that **he** had a positive…”). In the coherent case pronouns do not need explicit identity processing. The language interpreter looks for a suitable mental representation that provides the referent, i.e., Ronald, for the pronoun. So, the “he” does not create a mental representation of a second person to be explicitly identified with Roland. However, for ambiguous and failing pronouns a new representation for the person designated by the pronoun is needed and leaves the reader wondering with who of the earlier mentioned people this person might be identical.

### Equations in the brain

Equations such as “4 × 2 = 8” have been extensively studied in number-processing and arithmetic-computation neuroscience. While no study has used a control condition that would allow identification of numerical identity processing, several studies have contrasted different types of number processing, which are more or less likely to elicit numerical identity processing. The left IPL, specifically the left AG, is more strongly activated during arithmetic fact retrieval (e.g. 2 × 4 = 8) compared to number magnitude processing (e.g. 43 – 12 = 31) (Chochon et al., 1999; Dehaene et al., 2003; Pletzer, 2016; Pletzer et al., 2011). Arithmetic fact retrieval typically refers to the retrieval of known identities involving single digit additions or multiplications as memorized in multiplication tables (e.g., 2 × 4 is 8). Whereas, magnitude processing consists of executing a computation that yields a result (e.g., 43 – 12 makes 31). Hence the reported activation differences could be due to processing identity or fact retrieval. However, the fact that left IPL is also more active during exact calculation than during approximation (Dehaene, 1999) cannot be attributed to fact retrieval, as it is involved in both tasks. Clearly, identity plays a role in exact calculation but not in approximation, which suggests that the left IPL is also involved in identity processing and not just fact retrieval. Interestingly, the IPL, specifically the SMG, is more strongly activated in adults, when judging the correctness of equations, than in adolescents (Rivera et al., 2005); suggesting a developmental specialization of this area for identity processing.

In sum, studies that contrast tasks of greater emphasis on numerical identity processes with tasks of lesser emphasis tend to activate the left IPL, which was also singled out as identity relevant in studies with identity statements (Arora et al., 2015) or failing anaphoric reference (Nieuwland et al., 2007). Since left IPL has been strongly associated with arithmetic fact retrieval (Dehaene et al., 2003) we have to take care, our activations of the left IPL cannot be attributed to retrieval effort.

Accordingly, we have identified identity processing as a potential domain-general candidate function to be subserved by hub areas and preliminary evidence shows that cognitive domains that require identity processing appear to overlap in hub-areas. In order to address this hypothesis more explicitly, we require an experimental design that allows us to isolate explicit identity processing from its linguistic or numerical context. To model the identity process within each domain, we contrast conditions that require explicit identity processing with conditions that only differ in the respect that they do not require explicit identity processing for both domains. Furthermore, to cleanly model the identity process irrespective of any domain-specificity, we assess the overlap between the identity contrasts for both tasks via a conjunction analysis. We then assess, whether areas of overlap lie within the s-core of hub areas, including the PC, PCC, IPL, SPL and paracentral lobe and also control whether additional identity-related activations can be found in other brain areas. If identity-related activations can be found specifically within areas known as hubs, identity processing may well be one of the domaingeneral cognitive functions that hubs provide to various domain-specific brain networks.

## Experimental Procedures

### Participants

Of 35 participants recruited, 33 participants’ (15 males) with an average age of 23.53 years (SD = 5.40) were included in the study. Two participants were excluded from the data analysis due to excessive head movement (> 4mm translation), and higher percentage of outlier voxels (ranged between 50–70% outlier voxels computed with the program *fsl_motion_outliers* using *“framewise displacement”* as metric). All participants were recruited from the university and university hospital clinic, and received course credit or small monetary reimbursement. They were all native German speakers, had normal or corrected-to-normal vision, and no history of psychological or neurological disorders. Written informed consent was obtained from all participants prior to scanning. The ethics committee of the University of Salzburg has approved the study.

### Design and stimuli

*Language tasks:* For language based identity statements (LANG) there were three conditions represented by vignettes consisting of 3 German sentences (see Table 1 for example). In each vignette the first sentence introduced two people, e.g., the dentist, and Lilli. The second sentence introduced a third person whose identity was under question (e.g., Mr. Dietrich whose bag was found, or to whom a letter needed delivering, etc.). The third sentence differed according to condition. In the *identity condition* (LANG. = ) it informed that the third person (Mr. Dietrich) was identical to one introduced before (the dentist). In the non-identity (LANG.≠) condition the third person was unambiguously a new person (Mrs. Dietrich visits the dentist) and not one of the people introduced before. Additional filler (LANG.f) trials were introduced, as closely matched controls but turned out to elicit unwanted identity thoughts as several participants reported that they thought Mr. Dietrich was the same person introduced as “the dentist”. Moreover, these trials are liable to yet another identity interpretation. Mention of the dentist and his office followed by Mr. Dietrich also being a dentist could lead to the interpretation that several dentists are working at this office and Mr. Dietrich is (identical with) one of them (Kamp and Reyle, 1993).

There were two sets of 45 different vignettes. Set 2 duplicates set 1 but with different names of people. Each vignette was adapted for each of the three conditions, resulting in 270 different stories. For a particular participant each vignette was used only once, that is, 30 trials per condition, 90 altogether.

Variation of sentence length across all vignettes and conditions was very small: The average number of words for the two context sentences was 14.46 (± 0.64), for the last condition sentence 5.26 (± 0.31). There is no significant difference among all stories for context sentences or condition sentence (both *p’s* ≥ 0.46). On 30% of the trials a forced choice comprehension questions about one of the persons in the vignette followed (for example see Table 1: “Who owns the bag: Dentist or Lilli?). This test was to ensure that participants were attentive during the task and were able to process information from all three sentences. The order of correct and incorrect options was counterbalanced across conditions. Overall accuracy was around 88%, indicating that participants were attentive and understood the task.

Functional neuroimaging was divided into two sessions. Each session had 45 trials, 15 trials percondition, and 15 comprehension questions. The order of sessions was counterbalanced across participants. Every trial started with the fixation-cross for 3500 ms, followed by the context sentences for 5500 ms, then the condition sentence for 4000 ms, and finally the test question was presented for 5000 ms. A single trial without questions lasted for 13 s, and with question 18 s. A single session took 11 min.

*Mathematics tasks:* For the mathematical equations (MATH) there were three conditions (see Table 2). In two of them two arithmetic formulae were presented simultaneously to the left and right side of the screen and participants were instructed to compute the result of each (see Table 2). In the *identity condition* (MATH. = ) both formulae had the same solution (e.g.: “3 × 8” and “36 – 12”), while in the *non-identity condition* (MATH.≠) they had different solutions (e.g.: “3 × 9” and “36 – 12”). Participants reported that maintaining and comparing two separate values in working memory in the MATH.≠ condition was more effortful compared to the MATH.= condition, where they were required to maintain only one value in working memory. Accordingly, an additional low effort-control condition (MATH.c) was introduced, in which only one equation was presented at a time.

Each condition consisted of 40 trials. The three conditions were matched for the difficulty of equations, including size of multiplications and subtractions, parity, divisibility by 5 or 10, borrowing, and decade distance in the subtraction items. Ties were excluded, i.e. all subtractions consisted of four different digits and all multiplications consisted of two different digits. To minimize effort in MATH.≠ compared to MATH. =, subtractions had solutions unrelated and decade inconsistent with the multiplication results (Domahs et al., 2007). In the MATH.≠ condition half of the multiplication results were larger the other half smaller than the subtraction results. On average, in the MATH.≠ condition the difference between the result of subtraction and multiplication was zero (SD = 10.70). For half of the items the larger result was presented in the right side of the screen, for the other half on the left.

All equations were presented for 7000 ms following a 3500 ms inter-stimulus interval during which the fixation cross was presented. On 30% of the trials participants were probed by a Yes/No question presented for 5000 ms (see Table 2). The overall accuracy on the math task of about 94% indicates that participants were attentive and followed the task.

Functional neuroimaging was divided into two sessions. Each session comprised 60 trials, i.e. 20 trials per-condition intermitted by 20 null events (fixation cross) of the same duration. The presentation order of sessions was counterbalanced across participants. Each session lasted for 15.5 min, and functional imaging of the entire math task took 31 min. In the training session participants were introduced to the language and the mathematical task. They were instructed to read and understand the sentences or carry out each calculation with care, so they can answer occasional test questions.

### fMRI data acquisition

Functional and structural images were acquired on a Siemens 3 Tesla Tim-Trio Scanner, located at the Chris tian–Doppler–Clinic, Salzburg. Functional images sensitive to the BOLD contrast was obtained with a T2*-weighted gradient echo-planar imaging (EPI) sequence using a 32 channel head coil. Per subject, two sessions of the language task with a total of 310 EPI images, and two sessions of the math task with a total of 430 EPI images, including six dummy scans at the beginning of the functional images were acquired to allow transient signals to diminish (TR = 2250 ms; TE = 30 ms; matrix size = 64 × 64; voxel size = 3.0 × 3.0 × 3.0 mm^3^; slice thickness = 3.0 mm; slice gap 0.3 mm; FOV = 192 mm; flip angle = 70°). Thirty-six axial slices were acquired parallel to the AC–PC line, covering 118.5 mm of the z-axis. FieldMap data were acquired for undistorting the EPI Sequences (TE = 4.49 ms/6.95 ms). In addition, a sagitally oriented high-resolution structural scan was acquired from each subject using a T1-weighted MP-RAGE sequence (GRAPPA PE = 2; TR = 2300 ms; TE = 2.94 ms; TI = 900 ms; FA = 9°; voxel size 1.0 × 1.0 × 1.0 mm^3^; 192 slices per volume).

### fMRI data analysis

Preprocessing and statistical data analysis was performed by Statistical Parametric Mapping software SPM12 (Wellcome Department of Cognitive Neurology, UK), implemented in MATLAB 8.1 [R2013a] (Matworks, Natick, MA, USA) runtime environment. As a first preprocessing step images were despiked using the 3dDespike option as implemented in afni^23^ to improve realignment. Realignment and unwarping making use of the fieldmap was performed in SPM12. For the identification and correction of non-physiological noise a biophysically-based model (Functional Image Artefact Correction Heuristic, FIACH, Tierney et al., 2016) was applied. Images were filtered and six regressors of physiological noise were extracted for later use in first-level models along-side the six realignment parameters. The filtered images then underwent the standard SPM12 pre-processing pipeline including slice time correction, co-registration of functional to structural images, segmentation of structural images using CAT12 and normalization of functional images to MNI space (Montreal Neurological Institute, McGill, Montreal, Canada) with isotropic 3 mm voxels. The normalized images were smoothed with 6 mm FWHM Gaussian kernel.

For statistical analyses a two-stage mixed effects model was applied. First-level analysis was performed separately for each task. In each task, one regressor was modelled for each condition (identity =, non-identity ≠, and control c). Both, language and equation trials were modelled as block. In language tasks, all trials of each condition were modelled along with the context sentence with 9.5 s duration. For the math tasks, all trials of each condition were modelled with 7 s duration. For both kinds of task, question events as well as the six realignment parameters and six FIACH parameters of physiological noise were modelled as regressors of no interest.

The low frequency noise was removed by high-pass filter with a cut-off of 128 s, and serial correlation was taken into account using an autocorrelation AR (1) model (Friston, 2002), as implemented in SPM12. For each task, one first-level contrast was defined comparing the identity condition to the control conditions. For the language task, this contrast comprised LANG. = > LANG.≠. The LANG.f condition’s filler trials were not used in group level analysis. For the math task, both controls were used to avoid effort-related confounds (MATH. = > MATH.≠ & MATH.c). Contrast images for both tasks were entered into a full factorial model at the second level including the factor task. Second level contrasts were defined for each task and their conjunction was evaluated (Friston et al., 2005; Nichols et al., 2005). In a first-step, ROI-based analyses using a mask for the s-core of hubs were performed to assess, whether overlapping activations can be found in hub regions. A mask was derived from the brainnetome atlas (Fan et al., 2016) including PC, Cuneus, PCC, IPL and SPL, representing the s-core as described in Hagmann et al. (2008). The brainnetome atlas defines areas based on their homogeneity in terms of functional connectivity to other brain areas and accordingly also allows a fine-grained analysis of sub-regions within hub-areas. In a second step, whole brain analyses were performed in order to explore, whether overlapping activations can also be found in other areas. For all statistical comparison we used a primary voxel-wise threshold of *p* < 0.001, and a cluster extent threshold of 20 voxels, along with secondary threshold of *p* < 0.05, corrected for multiple comparison using family wise error (FWE) at the cluster-level.

## Results

### ROI-based analysis – are hub areas involved in domain-general identity processing?

*Language task:* The identity condition showed significantly stronger activation compared to the non-identity (LANG. = > LANG.≠) condition in the bilateral IPL, the PC, and in the PCC (Table 3).

*Math task:* In the mathematics task, the identity condition (MATH.= ) was contrasted against the non-identity (MATH.≠) and the effort-control (MATH.c) condition. Like in the language task, significant activations were observed in the left IPL, the PC and in the PCC (Table 3).

**Fig. 1:**
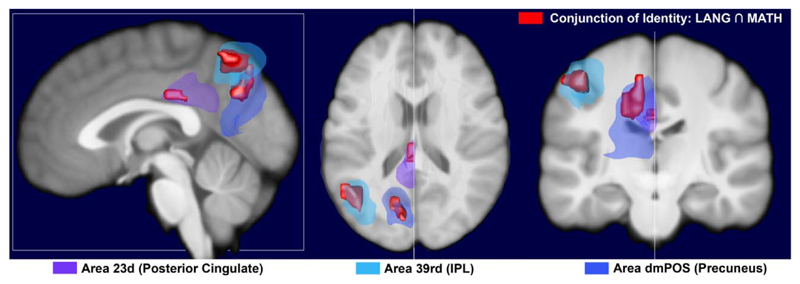
Conjunction activation of language and math identity (red) in relation to subregions of the brainnetome atlas: *A39rd* – rostrodorsal area of Inferior Parietal Lobule (light blue); *dmPOS* – dorsomedial parietooccipital sulcus – a subregion of Precuneus (blue); *A32d* – dorsal area of Posterior Cingulate Cortex (purple). All clusters were superimposed by the Scalable Brain Atlas (SBA) Composer on an average template of 40 T1 images from the Human Connectome Project. The activation clusters were thresholded at *p* < 0.05 FWE and masked with the s-core of hub areas. See Video S1 in Supplementary material for a 360° view of theses clusters. (For interpretation of the references to color in this figure legend, the reader is referred to the web version of this article.)

*Conjunction:* The conjunction analysis revealed that the identity contrasts showed consistent activation across both tasks in the left IPL, PC, and PCC (Table 3). A subregion analysis using the brainnetome atlas revealed that these clusters overlap to a large extent with subregions in the rostro-dorsal part of the IPL (39rd), dorsomedial part of the Precuneus (dmPOS) and dorsal part of the PCC (23d) (Fig. 1).

### Exploratory whole brain analysis – are other brain areas also involved in domain-general identity processing?

Conjunction analysis at the whole brain level revealed no additional areas of overlap between the language based and math-based identity contrasts.

## Discussion

The purpose of the present study was to evaluate, whether brain areas interconnecting domain-specific networks, i.e., so called hubs, also fulfill some domain-general cognitive functions, that are relevant to several cognitive domains and can thus be accessed via several networks if required. We have outlined in the introduction why identity processing could well be an example of such a domain-general cognitive function and have developed a task design that allows us to isolate the identity process from any domain-specific contents via overlapping activation contrasts of a linguistic and a numerical task. If hub-areas were to subserve among others, identity processing, our hypothesis was that such overlapping activations could be found specifically within hub-areas and not elsewhere in the brain.

Indeed, our central result is that the conjunction of identity-related activations between the linguistic and numerical task, falls within the left IPL, the PC, and the posterior cingulate gyrus. These areas have not only been described as hubs of the rich club (van den Heuvel and Sporns, 2011), but as part of the s-core of the cortex (Hagmann et al., 2008), thus connecting to all other areas of the brain. No areas of overlapping activations were observed elsewhere in the brain, suggesting that – within the boundaries of statistical uncertainty – identity activations are hub-specific and concern in particular parietal hubs.

This conjunction of activations is in perfect agreement with the existing literature. All studies with contrasts sensitive to identity reported peak (or subpeak) voxels in the left IPL and many in the PC. In the overview Table A.1 in the Appendix, we summarize the activation peaks closest to our conjunction peak. All three studies with linguistic material (identity statements or anaphoric reference) showed peak activations in close vicinity to our conjunction peaks in the left IPL and the PC. Furthermore, the geometric centre of activation peaks for numerical contrasts that – in our opinion – are identity related, were in close vicinity to our conjunction peak in the left IPL. Those include studies that contrast (i) fact retrieval with magnitude processing (Delazer et al., 2003; Tschentscher and Hauk, 2014; Sammer et al., 2007; Yang et al., 2017; Rickard et al., 2000; Menon et al., 2000; Grabner et al., 2007; Pletzer, 2016), (ii) exact versus approximate calculation (Dehaene et al., 1999), or (iii) selection of a correct value in contrast to a control (Kong et al., 2005; Yang et al., 2017; Price and Ansari, 2011; Pletzer, 2016).

An important further question concerns the generality of our findings: Do all networks that have to deal with identity problems make use of the neural structures we have identified? To explore this question, we compare activation peaks from domains that require identity processing to the conjunction peaks identified in the present study (see Table A.1). These domains include: (i) recognition memory, (ii) false belief and (iii) visual perspective taking.

In order to better see the connection among these different areas we use mental files theory (Recanati, 2012). A mental file is a mental process that represents or refers to particular entity (its referent). Its function is to track its referent over time and collect information about it. Of particular interest are cases where two files are deployed for the same referent. Such coreferential files create an identity problem. The existence of two files makes one think of two entities unless one has identity information and can link the two files to make clear that they refer to only one entity. This process of linking coreferential files is common to the different fields under consideration.

Linking of files is required for recognition memory. A mental file for the item deployed at learning has to be linked with the mental file deployed during recognition. This distinction has been investigated with know/remember judgements (Tulving, 1985). The Appendix shows the activation peaks from two metaanalyses on recognition memory (Spaniol et al. (2009)) for left IPL and PC, which are indeed in the vicinity of our conjunction peaks. Furthermore, a recent study by Tholen et al. (2019) investigated re-identification of faces. The peak activations for identifying identical persons closely match our conjunction peaks (see Table A.1).

A most stunning overlap of our results exists with the Parietal Memory Network (PMN, Gilmore et al., 2015), which touches the default mode network (DMN) but has been established as an independent network (Hu et al., 2016; Yang et al., 2014). The overlap is near perfect: The centre points of its three constituent areas (Nelson et al., 2013) are each within less than 8 mm of the peak voxels of our three overlap areas. This network is responsive to quite intricate memory manipulations. For instance, all three areas are deactivated relative to baseline at initial encoding but active above baseline at retrieval (*encoding/retrieval flip*). Moreover, repeated encoding leads to increasing activation with the number of repeats (*repetition enhancement*). These characteristics raise the challenging question why our identity contrasts activate the same network. We briefly outline how identity processes might be involved in these memory activations.

As we have seen, recognition of study items at test require identity judgments (linking of coreferential files). The activation pattern characteristic of PMN may result from a difference in the number of elicited identity judgments. This is not implausible. Nelson et al. (2013) used paired associate learning (Study: Service – Smile, Test: Service – ?). Taken as an identity processor PMN is at baseline constantly on the lookout for items identical to already known items. At initial encoding study items tend to be clearly novel. This results in immediate deployment of a new file and briefly frees PMN from looking for a known identity. It therefore deactivates. When later the same item is encountered at test a new file is deployed for it as the item presented at test. To recognise it as one that had been presented at study one has to pass an identity judgement, i.e., link the two files, in order to get the information “Smiles” from the original file for the test response. The linking activates PMN. Hence, all put together, the theory predicts deactivation at encoding and activation at test: the *encoding/retrieval flip.* Furthermore, participants may be aware of the same items having been presented more than once. In that case they entertain a corresponding number of coreferential files in need of linking. This increase of linkings with the number of study repetitions can account for the *repetition enhancement* effect.

These largely speculative points are to show that there might well be common processes that underlie memory performance as well as identity judgments, which explain why these different tasks activate PMN and produce the unusual activation patterns.

A perhaps more surprising need for identity computations exists for processing perspective differences, as in some theory of mind tasks. Attributing false beliefs are the best investigated cases (Saxe and Kanwisher, 2003). False beliefs about an object can be captured by a coreferential (vicarious) file for that object (e.g., that shows the object in a different location than where it really is). The file is associated with the other person (Perner and Brandl, 2005; Recanati, 2012) and is used vicariously for predicting other’s action. This requires linking of the vicarious file with one’s own regular file to understand that the other’s false belief is about the same object as one’s own belief, or else it would be understood as other’s belief about a different object. Linking of coreferential files is not required in theory of mind tasks generally unless perspective differences are involved as is the case in false belief tasks. The metaanalysis by Schurz et al. (2014) showed that among different theory of mind tasks only false belief tasks activated the dorsal part of the left IPL. The local peak in this area of the left IPL as well as the peak in PC is very close to our respective conjunction peaks (see Table A.1 in Appendix).

Another relevant area is visual perspective taking, which is the ability to recognize objects as identical, when presented from another viewpoint. The Appendix shows the peaks from an overlap analysis between a metaanalysis of false belief and visual perspective (Arora et al., 2017). The conjunction peak from the metaanalysis was very close to our conjunction peak in the left IPL and the same held true for the PC. A study used false sign vignettes (Perner et al., 2006). Since signs are not mental states those vignettes should not activate theory of mind areas but should activate areas sensitive to perspective differences. The peaks in Table A.1 confirm this expectation.

Also metaanalytic overlap has been found for Episodic Memory (remember/know) and false belief by Andrews-Hanna et al. (2014, Fig. 1). Their figure shows three areas of overlap, two in left IPL and one in the PC. Since no overlap peaks were reported, the Table A.1 in Appendix shows the peaks for each domain separately, which are still close to our conjunction peaks. Arora et al. (2015) looked for overlap between metaanalyses of episodic memory, false belief, and visual perspective taking. Peaks of overlap of all three areas were found in the left IPL and PC, both in close vicinity of our conjunction peak (see Table A.1). With special attention to the left IPL, Humphreys and Lambon Ralph (2015, Figs. 1 and 3) reported metaanalytic overlap of activations in the angular gyrus (with some activation in SMG) for numerical fact retrieval, episodic retrieval, and also sentence level semantic processing with a peak close to our conjunction peak (see Table A.1). Noonan et al. (2013) provide a metaanalysis of high versus low semantic control studies with a peak in the left IPL (dorsal angular gyrus). Semantic control examples, e.g., homonyms and metaphors, have a close affinity to identity as they involve semantic ambiguity. In contrast to identity statements, which clarify that two expressions have the same referent, homonyms or metaphors suggest a particular referent when in fact a different one is meant. No other language regions were activated by the identity condition over the control condition as both conditions require processing of linguistic information.

In summary, the overview table in the Appendix shows in an impressive manner that all existing studies that involve identity processes report activation in the left IPL in close vicinity to our conjunction area. This strongly suggests that the left IPL harbors metacognitive expertise that is consulted by several networks in need of identity processing. These are identity statements, equations, recognition, semantic control tasks, and various perspective tasks. This result is important for findings in other disciplines, notably cognitive development. Tasks that require identity processing in almost all the mentioned domains appear to be mastered by children around 4 years and correlate specifically with versions of the false belief task (Perner and Roessler, 2012 for review). Before passing the false belief task children fail to (1) profit from identity statements (Perner et al., 2011), (2) flexibly deal with alternative names for an object (Doherty and Perner, 1998; Doherty, 2000; Perner et al., 2002), (3) understand homonymy (Doherty, 2000), (4) appreciate ambiguous figures tasks (Doherty and Wimmer, 2005; Wimmer and Doherty, 2011). (5) Level 2 visual perspective tasks (how different people can see an object differently) but pass Level 1 (which objects different people can see) (Hamilton et al., 2009), (6) false sign tasks (Perner and Leekam, 2008), and (7) appearance-reality tasks (Gopnik and Astington, 1988; Taylor and Carlson, 1997; Courtin and Melot, 2005). Before that age they also fail to (8) understand equinumerosity (Sarnecka and Wright, 2013). This is an impressive developmental concordance across different domains, difficult to explain if separate networks are responsible for each domain. Our finding and our review of existing findings provides an answer: Changes in the neural processors in the left IPL time the performance on identity problems across different domains.

Whereas the dorsal left IPL is consistently activated when identity processing is required, the evidence for the PC is less striking. There are several gaps in the table, where relevant studies did not report any PC activation. Moreover, the peaks from the literature spread much more widely around our conjunction peak in the PC than in IPL, although this may simply be owed to the fact that activation clusters in the PC tend to be much larger than those in the IPL and peaks therefore more widely spread. Nevertheless, we cannot make any strong claims about how PC relates to identity processing. A plausible role as a member of the rich club hubs (van den Heuvel and Sporns, 2011) it may be needed to link the left IPL to where identity processes are needed.

Finally, our third conjunction area is located in the dorsal part of the PCC. Interestingly, the posterior cingulate is thought to play an important role in self-directed thought (Brewer et al., 2013) and in particular its dorsal part is “important for regulating the balance between internal and external attentional focus (Leech and Sharp, 2014, p. 24). This function dovetails nicely with the observation in our introduction that identity information causes a switch from information about the worldly objects to information about how objects are internally conceived. This suggests a tentative functional relationship between our three conjunction areas. Recognising identity in the left IPL leads to—at least temporary—refocusing from the subject matter to one’s subjective view. This activates the dorsal PCC via the PC. This explanation, however, leaves the question of why the dorsal PCC activation was only found in the present study and not in any other study listed in the Appendix. A possible reason for this may be the fact that most studies did not head on focus on contrasting identity with a control. That contrast was just one among others captured by these studies, which deprived them of sufficient power to detect the PCC activation. The one study that should not fall under this description is study 2 and 3 by Arora et al. (2014), which employed very similar contrasts as in our identity statements. Interestingly, at lower threshold both studies showed the PCC activations (study 2 peak: –9, –25, 31; study 3 peak: 0, –28, 31) both close to our conjunction PCC peak (*d* ≤ 9.4 mm).

The idea, that the IPL draws upon the PCC to switch attention from objects and persons in the world to one’s subjective view of them, is also supported by a more fine-grained analysis of our overlapping activations, using the brainnetome atlas (Fan et al., 2016). This probabilistic atlas specifically defines subregions with homogenous connectivity profiles based on structural and functional connectivity.

Our analyses show that the identity-related activations largely fall within subregions 39rd of the IPL, dmPOS of the PC and area 23d of the PCC. Structural connectivity patterns mapped within this atlas, show that area A23d of the PCC (with its centre at: –7, –23, 41) has no direct connection to the left IPL, but is connected to area dmPOS (–12, –67, 25) in the PC, which in turn connects to area A39rd (–38, –61, 46) matching our conjunction area in the left IPL. Metaanalysis based coactivation analyses show that these three areas are frequently coactivated in neuroimaging studies. This connectivity pattern appears to assign a more connecting function to the PC, allowing the IPL to access expertise located in the PCC or vice versa. This view of a connecting functions of the PC is in good agreement with (i) the fact, that PC and PCC activations are less consistently reported in specific association with identity processing in the literature, and (ii) the fact that the PC is among the cortical hubs showing the highest rich club centrality (van den Heuvel and Sporns, 2011).

Our finding’s main contribution to cognitive neuroscience is to substantiate one possible way how hubs may serve a cognitive function. Hubs are defined by their stronger structural and resting state functional connections with other parts of the brain and their activation by different domains. How the different domain specific activations interact is not yet fully understood (Humphreys and Lambon Ralph, 2015, 2017). Our data suggest that for the dorsal part of the left IPL in connection with the PC and PCC, the function is to provide metacognitive expertise to a variety of cognitive domains in need of such expertise. It remains to be explored whether other hubs, particularly hubs outside the parietal cortex, subserve other domain-general functions.

*Ethical approval:* All procedures performed in studies involving human participants were in accordance with the ethical standards of the University of Salzburg ethics committee.

## Supplementary Material

Appendix

## Figures and Tables

**Table 1:** Example of experimental trials of the language tasks

Conditions	Context sentences (5.5 s)	Condition sentence (4 s)	Test question + answers (5 s)
Identity LANG. =	The dentist goes to his clinic Lilli finds Mr. Dietrich’s bag	Mr. Dietrich is the dentist	Who owns the bag?” Mr. Dietrich /Lilli
Non-identity LANG.≠	The dentist goes to his clinic Lilli finds Mrs. Dietrich’s bag	Mrs. Dietrich visits the dentist	Who owns the bag?” Mrs. Dietrich /Lilli
Control LANG.f	The dentist goes to his clinic Lilli finds Mr. Dietrich’s bag	Mr. Dietrich is also a dentist	Who owns the bag?” Mr. Dietrich /Lilli

*Note:* The test question was only asked on about every third trial and served to check whether participants had paid attention to the text.

**Table 2:** Example of experimental trials of mathematics tasks

Conditions	Mathematical Equations (7 s)		Yes/No Question? (5 s)
Identity (MATH. = )	3 × 8	36–12	Are both results greater/smaller than 20?
			Yes/No
Non-identity (MATH.≠)	23–8	3 × 8	Are both results greater/smaller than 20?
			Yes/No
Effort-control (MATH.c)	23–8		Is the result greater/smaller than 20?
			Yes/No

*Note:* Yes/No questions are translated from German. The test question was only asked on about every third trial and served to check whether participants had computed the results of all formula.

**Table 3:** Brain activation of language, math, and conjunction of identity conditions

Region	*H*	*k*	*T*	Cluster peak MNI coordinates			*p*_FWE_
				*x*	*y*	*z*	
*LANG: Identity vs. Non-Identity*							
IPL	L	129	5.16	–39	–58	43	<0.001
IPL	R	45	4.15	45	–55	49	0.011
Precuneus	L	57	4.86	–12	–67	34	0.004
PCC	L	106	5.85	–3	–22	31	<0.001
*MATH: Identity vs. Non-Identity/Control*							
IPL	L	295	6.43	–30	–64	49	<0.001
	L	33	5.79	–27	–82	19	0.030
Precuneus	R	47	5.07	15	–67	37	0.009
PCC	L	33	4.53	–3	–22	28	0.030
*Conjunction: LANG ∩ MATH*							
IPL (area 39rd)	L	72	4.84	–39	–55	43	0.002
Precuneus (dmPOS)	L	51	4.86	–12	–67	34	0.007
PCC (area 23d)	L	29	4.53	–3	–22	28	0.043

*Note:* Significant clusters are reported at *p* < 0.05 FWE cluster level corrected and masked with hub areas of s-core as described in Hagmann et al. (2008).

## Footnotes

1 This is not the only method. It is used in symbolic AI and in language (with a heavy dose of contextual constraints). In connectionist systems and deep learning identity is captured by sameness of activation pattern.

## Abbreviations

AC–PC line: anterior commissure to posterior commissure
AR: autoregressive
EPI: echo-planar imaging
FA: flip angle
FIACH: Functional Image Artefact Correction Heuristic
FOV: field of view
GRAPPA: Generalized Autocalibrating Partial Parallel Acquisition
MP-RAGE: Magnetization Prepared - Rapid Gradient Echo
PE: phase encoding
SPL: superior parietal lobe
TI: inversion time
TR: time to response
TE: time to echo
