# Supplementary material for: What’s in a Hub?—Representing Identity in Language and Mathematics
Source: Neuroscience. Author manuscript; Available in PMC 2020 Mar 27. (PMC7100012; doi:10.1016/j.neuroscience.2020.02.032)
Supplement: Appendix [file EMS86023-supplement-Appendix.pdf]

APPENDIX A

See Table A.1

APPENDIX B. SUPPLEMENTARY DATA

Supplementary data to this article can be found online at https://doi.org/10.1016/j.neuroscience.2020.02.032.

Table A1. Comparison of the left IPL and precuneus conjunction peak with peaks or subpeaks from other studies

			Left IPL: supramarginal/ angular					Precuneus				
Topic	Study	content	*x*	*y*	*z*	*k*	*d*	*x*	*y*	*z*	*k*	*d*
Identity	Present study	Language ∩ Mathematics	−39	−55	43	75	–	−12	−67	34	58	–
Identity statements	Arora et al. (2015)	Identity Study 2	−54	−52	43	–	15.3	–	–	–	–	
		Identity Study 3	−39	−46	43	67	9.0	−12	−67	28	88	6.0
	Nieuwland et al. (2007)	Anaphoric reference	−40	−60	50	762	8.7	−4	−64	46	474	20.2
Equations	Geometric centre of all 18 sub- and peaks of 13 studies	Identity processing: likely > less likely	−37	−61	34		11.0	8	−48	21	n = 2	30.4
Episodic memory (EM)	Spaniol et al. (2009)	Meta-analysis: Subjective recollection	−54	−54	38	–	15.8	−6	−56	18	–	20.3
	Andrews-Hanna et al. (2014)	Episodic memory	−48	−42	52	170	18.1	−4	−54	24	1110	18.2
	Tholen et al. (unpublished)	Face re-identification	−36	−58	40	–	5.9	−12	−73	43	–	10.9
Perspective	Schurz et al. (2014)	FB Metaanalysis	−44	−61	40	–	8.4	0	−62	33	–	13.0
	Perner et al. (2006)	False Signs	−42	−63	36	–	11.0	–	–	–	–	
	Andrews-Hanna et al. (2014)	Mentalizing	−46	−60	30	1169	15.5	4	−54	32	2487	20.7
	Biervoye et al. (2016)	Problems with belief Focal point of lesion	−40	−68	34	69	15.7	n.a.				
Meta-analytic overlap	Arora et al. (2015)	EM ∩ FB ∩ vPT	−41	−61	40	19	7.0	6	−51	45	2	26.4
	Humphreys and Lambon Ralph (2015)	EM ∩ numerical fact retrieval ∩ semantics	−48	−64	34	–	15.58	n.a.				
	Noonan et al. (2013)	Metaanalysis executive semantics	−41	−55	45	58	2.8	n.a.				

Note: k = cluster extent in voxel; d = Euclidean distance between the studies' peak and the conjunction peak of the present study; n = number sub-peaks; EM: Episodic memory; FB: False belief; vPT: Visual Perspective Taking.

(Received 2 April 2019, Accepted 18 February 2020)
(Available online 27 February 2020)
